# Diagnostic Value of Low-Dose 256-Slice Spiral CT Angiography, MR Angiography, and 3D-DSA in Cerebral Aneurysms

**DOI:** 10.1155/2020/8536471

**Published:** 2020-01-13

**Authors:** Yan Feng, Sheng Jie Shu

**Affiliations:** Second Affiliated Hospital of Harbin Medical University, China

## Abstract

**Objective:**

To evaluate the diagnostic value of low-dose 256-slice CT angiography (CTA), MR angiography (MRA), and three dimensional-digital subtraction angiography (3D-DSA) in cerebral aneurysms.

**Methods:**

CTA, MRA, and 3D-DSA were performed in all enrolled patients to explore the diagnostic significance of the three different examinations.

**Results:**

There were 92 aneurysms confirmed during DSA and surgery in 79 patients. The diagnostic coincidence rates of DSA, CTA, and MRA were 96.7%, 89.1%, and 86%, respectively. The diagnostic coincidence rates of CTA and MRA were lower than those of DSA. The detection rate of CTA for aneurysms less than 3 mm in diameter was higher than that of MRA.

**Conclusion:**

CTA, DSA, and MRA have their own advantages and disadvantages in the diagnosis of cerebral aneurysms. They all have the diagnostic value for aneurysms.

## 1. Introduction

At present, the three dimensional-digital subtraction angiography (3D-DSA) is the gold standard for diagnosis and evaluation of cerebral aneurysms. But it is an invasive examination with risks [1]. Other timely and safe clinical diagnostic tools still need to improve the prognosis of patients under emergent conditions. With the development of medical science and technology, CT angiography (CTA) and MR angiography (MRA) have become important methods for the diagnosis and treatment of cerebrovascular diseases. These methods are minimally invasive or noninvasive, time-saving, and low-cost and produce high-quality images. However, the clinical effects reported in some studies are quite different [2, 3].

In this study, we compared and analyzed the diagnostic results of DSA, CTA, and MRA for intracranial aneurysms and discussed their advantages and disadvantages in order to help clinicians choose the appropriate diagnostic methods.

## 2. Material and Methods

100 patients suspected of having intracranial aneurysms were admitted to the Second Affiliated Hospital of Harbin Medical University from February 2014 to June 2019 (the study period). Seventy-nine patients with 92 intracranial aneurysms confirmed during DSA or surgery were retrieved. All patients included in this study received DSA, CTA, and MRA. The patients and their families were informed of the study and signed the informed consent form.

The inclusion criteria were patients with cerebral aneurysm. The exclusion criteria of the patients are as follows: (1) allergic to contrast media; (2) with history of vascular interventional embolization; (3) with severe diabetes mellitus or hypertension; (4) with severe abnormal liver or kidney function; (5) with other malignant tumors; (6) who were breast-feeding or with pregnancy; (7) with mental disorders; and (8) with history of craniotomy.

The 3D-DSA was performed using a Philips Allura Xper FD 20 X-ray system, and the contrast agent was iohexol (300 mgI/ml). The Seldinger method [4–8] was used to puncture the right femoral artery. After a successful puncture, a catheter sheath was implanted. Heparin was routinely used for anticoagulation, and a contrast medium was injected into the opening of the bilateral internal carotid artery and vertebral artery for cerebral angiography. The total injection volume of the contrast medium was 6-7 ml, at 3-4 ml/s. During the course of angiography, a 258-degree complex trajectory rotation scanning was performed. All patients received 3D-DSA under general anesthesia.

The equipment for the MRA examination was the GE Discovery MR 7503.0T nuclear magnetic resonance scanner. Time-lapse magnetic resonance angiography (TOF-MRA) and the standard head coil were selected. The transverse and sagittal positions were double positioned. The transverse and sagittal T1WI and T2WI scans were performed. The interval between layers was 0.1-1.2 mm and the thickness of layers was 5-6 mm. Scanning range included the internal carotid artery intracranial segment; the cerebral artery ring and the anterior and middle cerebral arteries and their main branches; the vertebral-basilar artery; and the posterior cerebral artery and its main branches.

The CTA examination was performed in a 256-row GE Revolution CT with the following scanning parameters: voltage 80KV, automatic milliampere, scanning layer thickness 0.625 mm, pitch 0.969 : 1, rotational speed 0.4 s/circle, and bed speed 19.37 mm/s. A routine craniocerebral plain scan was performed first, then a 40 ml contrast agent (20 ml contrast agent + 20 ml saline) was injected slowly at the speed of 4.5-5.0 ml/s, and a bilateral carotid artery filling time-density curve was monitored at the level of C4 to calculate the optimal delay time. The head scan was performed from the skull base to the top of the skull by plain scan + enhanced double − phase scanning; meanwhile, the venous channel was established and then high pressure was applied. Iopromide (Ultravist 60 ml) was injected through the elbow vein at a rate of 4.5-5.0 ml/s, and then 30-40 ml saline was injected at the same rate.

The original data of 3D-DSA was transmitted to the sys-workplace workstation and processed by VR technology. The original data of CTA was reconstructed by the machine-borne iterative reconstruction algorithm ASIR-V technology and transmitted to the GE ADW4.7 workstation. For those who still have artifacts in the CTA, the mathematical model of CT simulation system of Harbin University of Technology is applied to remove artifacts. Then, the image was silhouetted, where the enhanced image was subtracted from the plain scan image, and the image was obtained by volume rendering (VR), multiplanar reconstruction (MPR), and maximum intensity projection (MIP). The original MRA data was reconstructed by maximum density projection (MIP) through the built-in operation software of the console, and the local MRA data is reconstructed according to the actual situation.

The imaging results of DSA, MRA, and CTA in patients with intracranial aneurysms were observed, and the detections of intracranial aneurysms by the three methods were compared. CTA and MRA images were independently interpreted by two experienced radiologists. The number of aneurysms detected was recorded, and the maximum diameter of aneurysms was measured. The images were classified according to the types of large aneurysms (10-25 mm), middle aneurysms (6-9 mm), small aneurysms (3-6 mm), and tiny aneurysms (<3 mm). DSA images were interpreted by an interventional radiologist and a neurointerventional physician. The interpretation records were the same as those of CTA according to the previous CTA images. Based on the diagnostic and surgical results of 3D-DSA, the clinical diagnostic efficacy of CTA and MRA was evaluated. All measurements are carried out on the postprocessing workstation, and the operator was allowed to rotate the image from any angle to obtain the best measurement angle and enlarge the image appropriately.

### 2.1. Statistical Analysis

Discrete data were expressed as number of cases (percentages) and were analyzed using a *χ*^2^ test or a Fisher exact test, whichever was applicable. Continuous data were shown as mean ± standard deviation (SD). SPSS24.0 (IBM Corp, Armonk, NY) was used for statistical analysis. A two-tailed *p* < 0.05 is considered significantly different.

## 3. Results

Seventy-nine patients with 92 intracranial aneurysms confirmed during DSA or surgery were retrieved. Among them, forty-one were males and 38 were females, aged from 29 to 71 (42.8 ± 7.9). Eight of the 79 patients had no obvious clinical symptoms, and the other 71 patients had sudden severe headache and vomiting as the first symptoms, including 35 cases of transient disturbance of consciousness, 5 cases of epilepsy, and 6 cases of psychiatric symptoms. Physical examination showed that 67 cases had a positive meningeal irritation sign, forty-one cases had optic disc edema, nine cases had fundus hemorrhage, and 19 cases had positive pathological symptoms.

Of all the 92 aneurysms, 67 were single aneurysms and 25 were multiple aneurysms. The diameter of aneurysms ranged from 1.2 mm to 21.5 mm, with an average of 5.8 mm. Among them, 17 (18.5%) aneurysms were less than 3 mm, 45 (48.9%) aneurysms were 3-6 mm, 17 (18.5%) aneurysms were 6-9 mm, and 13 (14.1%) aneurysms were more than 9 mm.

In this study, among the 92 aneurysms, there were 21 anterior communicating aneurysms, 9 posterior communicating aneurysms, 3 anterior cerebral aneurysms, 27 middle cerebral aneurysms, 1 posterior cerebral aneurysm, 29 internal carotid aneurysms, and 2 vertebrobasilar aneurysms.

The diagnostic coincidence rates of DSA, CTA, and MRA were 96.7% (89/92), 89.1% (82/92), and 86% (75/92), respectively. The diagnostic coincidence rates of CTA and MRA were lower than that of DSA. There was no significant difference between CTA and DSA, or between CTA and MRA (*p* > 0.05), but between MRA and DSA (*p* < 0.05) (Table 1). DSA was more sensitive than the other two methods, whereas CTA was more sensitive than MRA.

**Figure 1:**
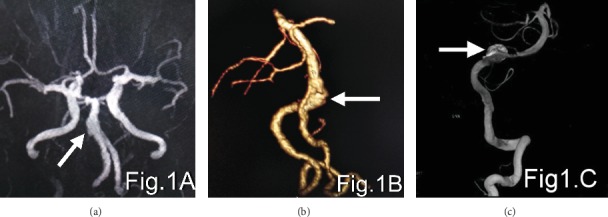
(a–c) show the MRA, CTA, and 3D-DSA, respectively, of basilar artery aneurysms in the same patient. The arrows show the aneurysm.

**Figure 2:**
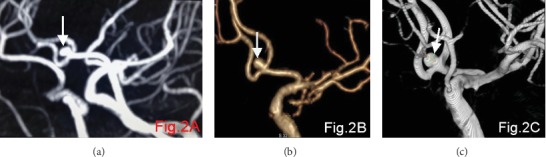
(a–c) show the MRA, CTA, and 3D-DSA, respectively, of the anterior communicating artery aneurysm in the same patient. The arrows show the aneurysm.

**Figure 3:**
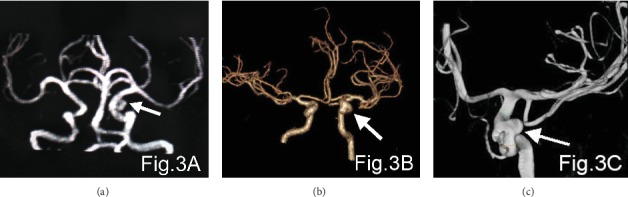
(a–c) show the MRA, CTA, and 3D-DSA, respectively, of the left internal carotid aneurysm in the same patient. The arrows show the aneurysm.

The detection rate of CTA for aneurysms less than 3 mm in diameter (76.5%) was higher than that of MRA (35.3%). The difference was statistically significant at *p* < 0.05 (Table 2). Figures 1–3 show the MRA, CTA, and 3D-DSA of a basilar artery, an anterior communicating artery, and a left carotid artery aneurysm in the same patient.

## 4. Discussion

DSA examination is regarded as the gold standard and a reliable and accurate diagnostic method. In this study, the sensitivity of DSA in the diagnosis of cerebral aneurysms is 96.7%. Compared with the traditional 2D-DSA, 3D-DSA has more advantages [9]. It can present stereoscopic images and rotate 360 degrees. It can observe the image of the lesion area in a three-dimensional space from any angle and present a comprehensive image. Anatomical connections of the aneurysm body, the neck, the parent artery, and the peripheral blood vessels are observed. In addition, small aneurysms concealed under the great artery and small lesions submerged in the skull base's bony structure can be found [10], which improves the diagnostic detection rate of aneurysms. However, DSA also has its drawbacks, such as iatrogenic damage to blood vessels, exposure to radiation, allergy to contrast agents, complications after angiography [11–13], and high diagnostic costs. Relevant studies have shown that the implementation of DSA for femoral artery catheterization will lead to increased risk of neurological complications in patients. If there is thrombosis, the development of aneurysms will be unclear and the probability of missed diagnosis will be increased [14, 15]. This restricts its clinical application. Therefore, it is very important to choose a simple, safe, and reliable examination method with high acceptance of patients after LAN clipping. In recent years, CTA, as a noninvasive and a rapid examination method, has been paid more and more attention in the diagnosis of aneurysms [16, 17].

In this study, patients with aneurysms were examined by CTA, and then CT simulation technology was used to remove artifacts. The effect was good, and the radial artifacts were significantly reduced. In the postprocessing step, by subtracting the corresponding pixel values, the signal-to-noise ratio (SNR) is reduced, and the high density of blood vessels is highlighted, which is more suitable for the display of diseased blood vessels [18].

On the basis of CTA, the subclinoid and intracranial internal carotid arteries can be observed by removing bone structure by software, and the resolution of distal small vessels can be improved [2]. In this study, the sensitivity of CTA for aneurysms was 91%, showing a high diagnostic value. CTA technology can obtain three-dimensional whole blood vessel data, which can simultaneously reflect arteriovenous lesions in three-dimensional space, and has a strong stereoscopic sense, which is conducive to the selection of aneurysm treatment options. The CTA imaging time is short, and scanning and postprocessing can be completed in a few minutes, saving time for the treatment of patients. The disadvantage is that there is still X-ray radiation, hence the need to inject contrast agent with allergic reactions [19].

Some studies [20] show that MRA imaging with a high field intensity (>1.5 T) is effective. In this study, the diagnostic sensitivity of MRA for aneurysms was 83.1%, which was lower than those of DSA and CTA. For larger aneurysms, the internal blood eddy in the aneurysms causes the loss of tumor signal, which has a negative impact on image quality. It cannot fully show [21] the relationship between the aneurysm neck and the parent artery, nor can it show the surrounding bone structure, wall calcification, and so on. In addition, the effect of background suppression is poor. Although MRA does not need injections of contrast agents, it takes a long time to examine and is contraindicated for those who have metal in their bodies. In this study, the main disadvantage of MRA is that the distal small vessels were easily saturated, which led to the loss of peripheral small vessels and affects the observation results. In addition, in some images the effect of background suppression was poor.

In this study, both CTA and MRA have high diagnostic efficiency for aneurysms, indicating that both methods can effectively detect aneurysms. We found that CTA has a high detection rate in aneurysms, which may be due to the removal of bone structure interference by CTA, leading to a higher vascular resolution and increased detection rate of intracranial aneurysms. In this study, DSA was more sensitive and accurate than CTA or MRA in the diagnosis of aneurysms. CTA was higher than MRA.

If the diameter of aneurysms is greater than 9 mm, all the three diagnostic methods can make accurate diagnosis and can clearly show the body and neck of aneurysms. If the diameter of aneurysms is between 3 mm and 9 mm, DSA has the best diagnostic effect, and CTA and MRA have a slightly poor diagnostic effect. For less than 3 mm, MRA is not good, detection is more difficult, and missed diagnosis is possible. The detection rate by CTA for aneurysms less than 3 mm in diameter was higher than that of MRA. It shows that CTA is superior to MRA in the detection of small aneurysms.

In conclusion, CTA, DSA, and MRA have diagnostic significance for intracranial aneurysms. They all have their own advantages and disadvantages. Therefore, in practical work, doctors should select suitable examination methods according to the actual situation of patients to ensure the accuracy of clinical diagnosis and improve the prognosis of patients.

## Figures and Tables

**Table 1:** Diagnostic results of CTA, MRA, and DSA in cerebral aneurysms (*n*, %).

Examination method	Positive (*n*)	Negative (*n*)	Specificity (%)	Sensitivity (%)
CTA	82	10	66.7	91.0
MRA	75	17	66.7	83.1
DSA	89	3	—	96.7

**Table 2:** Comparison of the coincidence of DSA, CTA, and MRA in detecting the size (maximum diameter) of aneurysms (*n*).

Inspection methods	Number of detections	≤3 mm	>3~6 mm	>6~9 mm	>9 mm
DSA	89	15	44	18	13
CTA	82	13	40	16	13
MRA	75	6	39	17	13

## Data Availability

The data used to support the findings of this study are available from the corresponding author upon request.
